# Community-funded integrated care outreach clinics as a capacity building strategy to expand access to health care in remote areas of Uganda

**DOI:** 10.1080/16549716.2021.1988280

**Published:** 2021-10-31

**Authors:** Rebecca G. Kinney, Henry Zakumumpa, Joseph Rujumba, Kevin Gibbons, Anna Heard, Omar Galárraga

**Affiliations:** a Health Access Connect, Colorado, USA; b School of Public Health, Makerere University, Kampala, Uganda; c Department of Paediatrics and Child Health, School of Medicine, Makerere University, Kampala, Uganda; d Health Access Connect, Kampala, Uganda; e Independent Consultant, Washington, DC, USA; f School of Public Health, Brown University, Providence, RI, USA

**Keywords:** Health equity, HIV, healthcare financing, Sub-Saharan Africa, community health planning

## Abstract

Most Ugandans live in rural, medically underserved communities where geography and poverty lead to reduced access to healthcare. We present a novel low-cost approach for supplemental primary care financing through 1) pooling community wealth to cover overhead costs for outreach clinic activities and 2) issuing microfinance loans to motorcycle taxi entrepreneurs to overcome gaps in access to transportation. The intervention described here, which leverages community participation as a means to extend the reach of government health service delivery, was developed and implemented by Health Access Connect (HAC), a non-governmental organization based in Uganda. HAC began its work in August 2015 in the Lake Victoria region and now serves over 40 sites in Uganda across 5 districts, helping government health-care workers to provide over 1,300 patient services per month (and over 35,000 since the program’s inception) with an average administrative cost of $6.24 per patient service in 2020. In this article, we demonstrate how integrated and appropriately resourced monthly outreach clinics, based on a microfinance-linked model of wealth pooling and government cooperation, can expand the capacity of government-provided healthcare to reach more patients living in remote communities. This scalable, sustainable, and flexible model is responsive to shifting needs of patients and health systems and presents an alternative approach to healthcare financing in low-resource settings. More rigorous evaluation of health outcomes stemming from such community-based models of service delivery is warranted.

## Background

Decreased proximity to urban centers leads to global inequities in health, education, and economic opportunities [1]. In Sub-Saharan Africa, transportation barriers limit care-seeking behavior and reduce access to HIV services, malaria treatment, and emergency obstetrical interventions [2–4] while long distance to the nearest health facility is a determinant of rural child mortality [5]. Round-trip transportation to a local health facility can be cost-prohibitive in settings with high poverty, and the resulting underutilization of health services is a critical global health issue [6,7].

Although close-to-community health services can overcome some of the barriers and improve health outcomes [8–10], such services are often constrained by time-bound, vertical project grants that support only a limited set of health interventions, poorly align with country priorities, and often create aid dependency [11–13]. Furthermore, many of the large aid organizations and their implementing partners work on independent budget cycles and are slow to distribute funding, which complicates efforts at strategic planning as well as timely project implementation [13]. Such volatile funding is especially detrimental for programs that serve people living with HIV and other chronic conditions, where reliable access to care and medicines is crucial to both disease and epidemic control [14].

A paradox emerges, however. As smaller, community-based care organizations seek to fill the gaps, they are constrained by regulations that prohibit patients from paying fees or pooling their wealth to improve access to government services. To be certain, the user fee debate is a complex and thorny one. Though early policies advocated for user fees as a strategy to improve quality, ration limited resources and discourage informal payment networks, many subsequent studies have demonstrated that user fees decrease health service utilization and negatively impact a wide range of outcomes ranging from health to household solvency to gender equality [15–19]. This has amplified a global movement for universal healthcare as a human right [20] – regardless of ability to pay – and has also put pressure on donor organizations, governments, and service providers to set policies requiring that health-care services are provided free-of-charge [16,17,21]. In the case of Uganda, user fees at public health facilities were abolished in March 2001, leading to increased utilization of health services [16]. However, what is often missing in this conversation is the fact that transportation costs can be *de facto* user fees, that lack of sufficient financing leads to decreased quality of care, and that replacing exorbitant travel costs with reasonable community contributions within a financially sustainable health service model may actually accelerate progress toward universal health coverage. In Mozambique, for example, transportation and other indirect costs were found to limit prenatal care for women even when the healthcare itself was free [22]. Indeed, the Ugandan example of eliminating user fees without acknowledging indirect costs such as lost wages or transportation compares unfavorably with government initiatives in other countries, such as Cambodia, where health equity funds embody a more holistic approach to financial risk associated with healthcare [17,18].

In this paper, we describe an intervention for expanding access to primary care for remote, underserved populations in the Lake Victoria region in Uganda through community-funded integrated care outreach clinics. We also use the successes and lessons from this model to advocate for a more nuanced approach to healthcare financing in low-resource settings that balances the obvious benefits of no-cost care with the long term benefits of predictable, efficiently distributed funding that is responsive to patient needs and prioritizes community engagement.

## Local setting

Uganda is a low-income country in Sub-Saharan East Africa composed of 135 districts with an estimated population of 45.7 million in 2020 [23]. In 2017, the maternal mortality ratio (MMR) was 375/100,000 live births and in 2018, the under-five mortality rate was 46/1,000 live births; on these metrics, Uganda outperforms Eastern and Southern Africa as a whole but compares unfavorably to global averages. [24]. Uganda is also at risk of failing to meet several of the Sustainable Development Goals (SDGs). According to a recent Voluntary National Review (VNR) Report, Uganda outperforms many other African countries – it is ranked 18^th^ out of 52 African countries on the metric of progress toward the SDGs – but the pace of change remains too slow to meet the 2030 deadline and is hampered by resource scarcity across multiple domains, including human resources for health, medical supplies, food, transportation, and quality primary education, among many others [25]. The MMR target of 70/100,000 by 2030, for example, would require an 80% reduction from the current level whereas the annual rate of decline has been about 3% per year since 2000 [26]. Furthermore, there are large urban-rural disparities in access to health services. A significant majority of Ugandans (86%) live rurally while only a minority (15–20%) of the country’s doctors work in rural areas [27]. The full-immunization rate for children aged 12 to 23 months is as low as 50% in rural areas compared with 61% in urban areas [28]. The consequences of under-immunization of children in Uganda, as for other countries, include not only higher morbidity and mortality but also lost economic opportunity: Gavi, the Vaccine Alliance, has demonstrated that vaccination is integral to achieving 14 of the 17 SDGs, and the return on investment of immunization in Gavi-supported countries, when considering costs averted plus broader societal value, is $54 per every $1 spent [29,30].

These lagging maternal and child health indicators underscore the challenge of accessing healthcare in Uganda. The Lake Victoria region in Uganda, where Health Access Connect (HAC) began its work in 2015, faces particularly daunting health challenges and barriers to care. HIV prevalence rates are 3 to 5 times the national average due to a combination of structural and psychosocial barriers (mobile lifestyle, irregular work hours, stigma), leading to overall decreased health-seeking behavior [31,32]. Furthermore, many communities in the region (especially island and shoreline communities) are inaccessible by car and have no regular mass transit (bus or ‘*matatu*’ van) options. Motorcycles, including motorcycle taxis or ‘*boda bodas*’ are the main form of transport for day-to-day activities as well as for health-related travel but are expensive. Subsidizing the cost can help: one study in another part of Uganda demonstrated that a *boda boda* voucher program improved women’s ability to reach emergency obstetrical and newborn care, demonstrating the potential utility of adapting this type of travel for healthcare [33]. Of course, relying on *boda bodas* for routine medical travel is not without risk. A recent study of road traffic injury (RTI) trends in Uganda found that *boda bodas* are responsible for more than half of all road traffic injuries in the country and consume a large proportion of the surgical budget at the Mulago National Referral Hospital [34]. Nonetheless, *boda bodas* fill an important gap in health-related transportation and HAC is always interested in exploring additional avenues to make transport safer and more accessible.

## Health Access Connect

HAC was conceived in 2014 to link remote communities to healthcare. While it originally focused on HIV service provision, HAC now works with the Ugandan government to identify underserved populations and establish a system to give trained government health-care workers the opportunity to provide a wide array of health services, including HIV testing, antiretroviral therapy (ART), malaria treatment, vaccines, family planning, perinatal and pediatric care, and blood pressure screening and treatment in remote, underserved communities.

## The intervention

**Figure 1.**
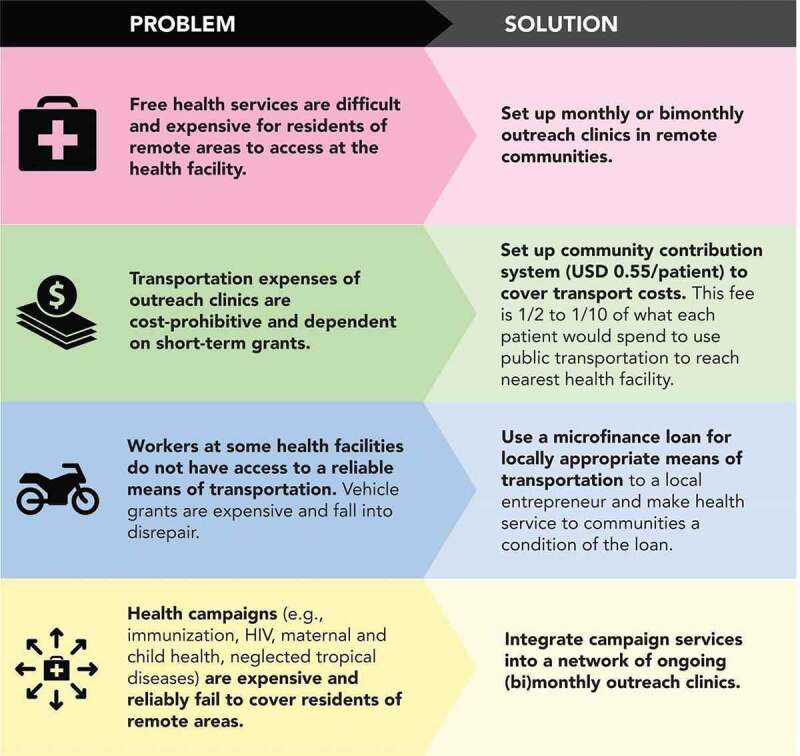
Health access connect pragmatic framework

HAC designed its intervention through consultation with government health-care workers. The HAC model works synergistically with government-financed healthcare to expand the existing capacity of the government health system and extend the reach of the care that the government is already providing for free. Following a pragmatic framework (Figure 1) to deliver necessary services to the most hard-to-reach populations, the model involves collaborating with trained frontline, government health-care workers (clinical officers, nurses, midwives, and, less often, doctors) and providing the government-mandated ‘safari day allowance’ (currently at least $4.60 per health-care worker per day) paid to health-care workers when they provide services outside their home facility.

The HAC model includes eight key elements

1. Geographic and census data and consultative interviews with key stakeholders at national, district, sub-county, and health facility levels are used to identify and select remote villages and target services.
2. Memoranda of understanding are signed with district local government units to set roles and with health facilities to commit health-care workers to serve at outreach clinics.
3. In selected remote communities that are over 5 km from the nearest health facility, a one-day outreach clinic occurs every 1 to 2 months.
4. As part of the wealth pooling strategy, a local community health-care worker or community leader collects a small fee from patients attending the clinic ($0.55 per patient) to cover transportation costs for health-care workers ($22–30 per outreach clinic day). The designated person then distributes the money for transportation, allowances, and mobilization. If the community wealth pooling does not collect enough money to pay for transportation, then HAC ‘tops up’ to ensure that service providers receive what they expect and that the program can maintain consistency of care.
5. All health services and medications are free and provided by public sector health-care workers.
6. At health facilities without a reliable form of transportation, HAC provides microfinancing for a motorcycle taxi to a local entrepreneur who then services outreach clinics as a condition of the loan. The favorable terms of the loan and lack of access to credit in rural areas make this an attractive offer. Entrepreneurs are chosen based on experience and endorsement by local leaders.
7. HAC’s primary roles are monitoring, data analysis, and ensuring continuity of the outreach clinics. The public sector health-care workers provide all services and necessary reporting to HAC and the national Health Management Information System.
8. Participation is voluntary, and some communities have decided not to accept the program. Patients can always choose to access services for free at the government health facility.

## Outputs

**Figure 2.**
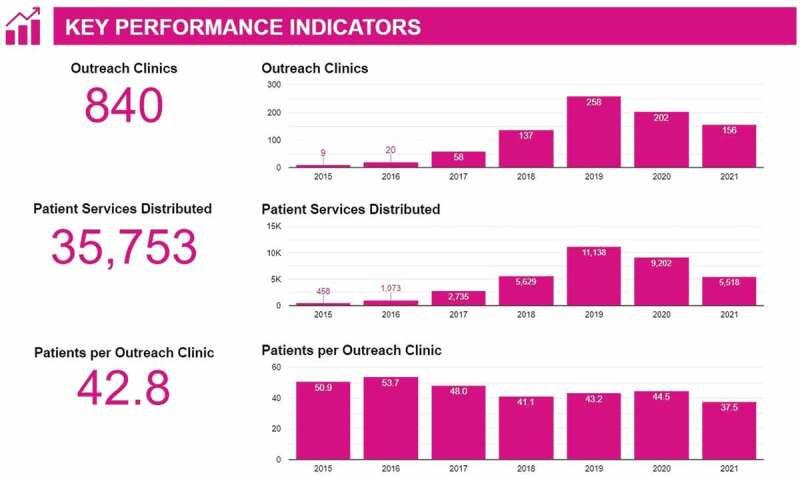
Key performance indicators, through June 2021

HAC oversaw its first outreach clinic in August 2015 and by the end of 2020, was operating in 49 sites across 5 districts in Uganda, providing over 1,300 patient services monthly. Between the program’s inception in August 2015 and June 2021, more than 35,000 patient services had been provided by government health-care workers in remote communities. Key performance indicators, such as number of patients per outreach clinic, number of couple years of (contraceptive) protection distributed per month, and number of patients on ART, are tracked over time using an interactive dashboard (Figure 2 and at https://healthaccessconnect.org/dashboards). HAC is keen to establish a more robust research agenda, once funding allows, that will include a more ambitious set of indicators to better measure the impact of community clinics on individual and community-wide health outcomes.

Field officers and management staff monitor outreach clinics remotely using phone calls, chat, and text messages with health-care workers and community members as well as through survey collection tools, such as OpenDataKit. HAC tracks service data through paper outreach clinic reporting forms, which health-care workers fill out and HAC staff enter into the database to generate analyses and reports.

Initially, the model’s goal was to expand access to ART in remote areas. Additional health services were integrated to allow people living with HIV (PLHIV) to maintain status confidentiality and soon the scope of service expanded further to serve the needs of more patients. Importantly, as the program has expanded its services, the administrative cost to HAC has remained low: in 2020, the program had an annual budget of $55,788, delivering services at an additional cost of approximately $6.24 per patient (Table 1). These expenses could feasibly be reduced if, at some point in the future, greater ownership of the model was assumed by the government, which already engages in similar administrative tasks. As an example, there are Chief Development Officers (CDOs) working with community groups at the district and subcounty administrative levels of the Ugandan government. In place of HAC, CDOs could liaise with the communities hosting health outreaches. There are District Health Officers already overseeing health services in a district, and they could assume HAC’s role in encouraging and sustaining the outreach clinics. These possibilities also underscore that HAC’s key ‘value add’ lies in coordinating, communicating, and connecting. In the absence of donor support, a motivated community with a responsive local government body could replicate the HAC model to extend the reach of government resources to remote communities.

**Table 1.** Growth of HAC and expenses between August 2015 and December 2020

Year	Outreach Clinics	Village Sites	Patient Services	Program Expenses	Additional expense per patient service
2015	9	3	458	$1,839	$4.02
2016	20	6	1,073	$2,952	$2.75
2017	59	9	2,765	$19,533	$7.06
2018	137	25	5,629	$26,912	$4.78
2019	235	43	11,138	$47,008	$4.22
2020	198*	49	8,946	$55,788	$6.24

*The decline in number of patient services delivered in 2020 was due to the Covid pandemic and associated public health restrictions across Uganda.

## Opportunities and barriers

The HAC model is attractive due to its creative mobilization of existing resources to enhance capacity, sustainable financing innovations, and emphasis on basic primary care services. These strengths offer opportunities for scaling to other similarly situated communities where geography constrains health-seeking behavior and where a microfinance-linked wealth pooling strategy could enable the delivery of essential, inexpensive health services that reduce or avert use of delayed services at higher, more costly levels in the health system.

In addition, the flexibility of the HAC model allows health-care workers to respond to the shifting needs of patients and the health system. Such adaptability has become a considerable asset over the past year when Covid-19 led to system-wide shutdowns in the delivery of care. In the beginning months of the pandemic, the number of outreach clinics and patient services provided decreased dramatically due to public health lockdown guidelines and uncertainty in the health-care sector. However, patient demand for community-based services subsequently rebounded and HAC increased outreach sites from 43 before the pandemic to 49 at the end of 2020. Especially in times of pandemic-induced social distancing, the HAC model seems fit-for-purpose: bringing care to patients in rural, low-density regions is clearly preferable to asking patients to travel long distances to a large central location. Given its resiliency and ability to sustain operations when so many other programs have had to shutter, the HAC model could also be leveraged as a primary partner in Covid-19 vaccine rollout and other health-care campaigns.

Even in typical times, integrated outreach clinics are a key component of healthcare provision in Uganda for maternal and child health services, including basic immunization packages, financed by primary healthcare (PHC) grants. While these funds are often delayed and are inadequate, they present an opportunity of pooling wealth at programmatic levels to better serve target communities. In the next phase, HAC will deepen engagement with district and health facility management to leverage additional resources from other programs, which may help to reduce both overhead costs and health service user contributions.

The provision of HIV care offers a glimpse of how deeper collaboration with government programming could work. The Ugandan Ministry of Health (MOH) is currently implementing a differentiated service delivery (DSD) model of antiretroviral therapy provision, which describes a client-centered approach to HIV care that aims to better serve the needs of PLHIV, maximize efficiencies in the health system, and improve client outcomes [35]. Because each country and community is unique, creating context-specific strategies is important but has proved challenging [36]. Uganda is currently implementing two broad categories of DSD models: i) Facility-based models and ii) Community-based models [37]. The community-based ART delivery models are: i) Community Drug Distribution Points (CDDP) and ii) Community Client-Led ART Delivery (CCLAD) [38]. The Ugandan Ministry of Health has set a target for providing over 25% of ART clients with community-based services, including enrolling 10% in Community Drug Distribution Point models and 15% in Community Client Led ART Delivery models (J. Kiggundu at AIDS Control Program of the Ministry of Health, personal communication with Kevin Gibbons, 7 April 2021), but as of December 2020, only 10.4% of classified active ART clients are receiving services under these models [39]. To reach these targets, the Ugandan government and its partners will need to identify ways to scale community-based DSD models. Given that HAC already engages in ART distribution to remote communities, the organization is well positioned to help fill the gap while also demonstrating a key benefit of public–private partnerships in this country where the private sector provides 70% of frontline services [40] and only about 30% of the total health expenditures are financed by government resources [41].

Even with these promising opportunities, HAC still faces challenges. The HAC operating model is sensitive to commodity stockouts as well as to patient volume – if too few patients attend an outreach and the wealth pooling fails to cover transport costs, there can be excess expenses that HAC grant funds must cover. Also, the HAC model depends on coordination with Ugandan government officials as well as the participation of the local community. This is a fundamental strength when it comes to aligning priorities and targeting the services that are in highest demand in selected communities. As noted above, it may also be a way to access co-financing from District Health Office PHC funds. However, the dependency is a potential liability if official priorities change or funding streams shift.

HAC’s wealth pooling strategy itself presents the greatest barrier to program expansion. HAC has been unable to partner with other synergistic organizations given Ugandan national, PEPFAR, and other international donor policies prohibiting any type of client contribution to access ART and other health services. While the wealth-pooling strategy is voluntary and invoked exclusively to cover the transportation expenses of health-care workers rather than to pay for commodities or services, and while the intervention has been shown to improve access and reduce costs to patients, the wealth pooling concept has been misinterpreted as a ‘user fee’ or ‘cost sharing’ to pay for free government health-care services, rather than a community initiative to bring health-care services closer to patients who reside far from the nearest health facility. Revisiting these financing regulations is critical, especially considering the aforementioned DSD targets and the inherent challenges in building enough government capacity to reach PLHIV in remote communities. Research is needed to better define and distinguish between different payment models so that the full financial risks associated with healthcare can be addressed. Nevertheless, the HAC model meets a critical need in the overcrowded global health arena: future evaluation focused on more rigorous investigation of patient outcomes, satisfaction with care, and changes in disease trends will improve the quality of HAC’s outreach model and facilitate translation of the model to other settings in our increasingly interconnected and changing world.

## Conclusion

Integrated and appropriately resourced monthly outreach clinics, based on a microfinance-linked model of wealth pooling and government cooperation, can expand the capacity of government-provided healthcare to reach more patients living in remote communities in a way that is sustainable. This promising implementation model should be more rigorously evaluated to assess health outcomes.

## Key findings

1. Low-cost, low-tech solutions can leverage existing community resources (e.g. public sector healthcare, community health-care workers, modes of transportation, and community wealth) to overcome geographic barriers to care.
2. Community engagement is essential: consistency of health service delivery builds patient trust, participation, and retention in care while coordination with government health leadership facilitates resource mobilization, sustainability, and ownership.
3. A more nuanced approach to healthcare financing that can accommodate wealth pooling and microfinance-linked solutions can help to lower transportation costs, expand capacity and extend quality of healthcare, and promote local entrepreneurship.

## Key implications

1. Local/regional policymakers, which include district-level officers and community leaders, should actively map and approach other service providers operating in the space for potential collaboration in order to pool resources at the partner level to better serve the community.
2. National stakeholders, which include various implementing organizations (IPs) as well as Ugandan government officials and the National SDG Taskforce, should push for further monitoring and evaluation of this approach to measure outcomes over time and adapt programs to different implementation contexts.
3. Global health stakeholders, which include international organizations (e.g. World Bank, World Health Organization) and large global health donors (e.g. Bill and Melinda Gates Foundation, Clinton Health Access Initiative, USAID, Global Fund, and Gavi), should continue to engage in robust but nuanced debate about how healthcare is accessed and financed, acknowledging that some flexibility will be required to reach remote populations in very poor settings.

## Paper context

In Uganda, as in many rural settings, access to healthcare is constrained by geography and poverty, leading to avoidable morbidity and mortality. Monthly integrated outreach clinics, predicated on a more nuanced approach to healthcare financing that can accommodate wealth pooling and microfinance-linked solutions, can lower transportation costs and expand the capacity of government-provided healthcare to reach more patients in a predictable, sustainable way that is responsive to patient needs and prioritizes community engagement.
